# Ascending stent in acute type A as a bridge to open repair

**DOI:** 10.1016/j.xjse.2025.100071

**Published:** 2025-09-10

**Authors:** Jason Ejimogu, Aakash Shah, Kimanthi Gicovi, Shahab Toursavadkohi, Mehrdad Ghoreishi

**Affiliations:** a Division of Cardiac Surgery, Department of Surgery, University of Maryland, Baltimore, Md; b Miami Cardiac and Vascular Institute, Baptist Health South Florida, Miami, Fla

**Figure:**
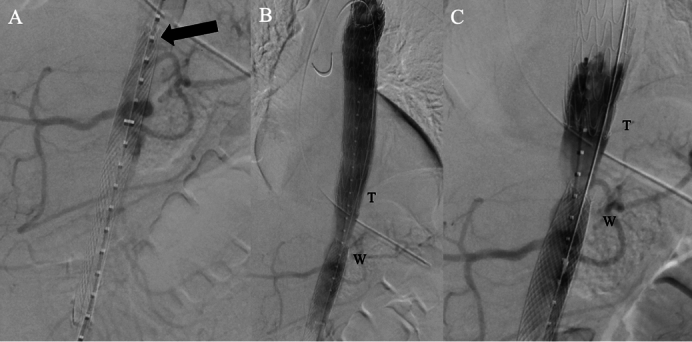
Aortogram of the descending thoracic aorta.

**Central Message**

An ascending aortic stent graft can temporize proximal entry tears in ATAAD with mesenteric malperfusion, safely bridging patients to delayed open repair.

Acute type A aortic dissection (ATAAD) is a life-threatening event with substantial in-hospital mortality, ranging from 7% to 30%.^1^ Patients with ATAAD who present with concomitant end-organ malperfusion and organ failure are at a significantly increased risk of postoperative mortality. Notably, mesenteric malperfusion syndrome has been reported to have a poor prognosis, with in-hospital mortality ranging from 60% to 75%.^2^ Emergency open aortic repair is recognized as the gold standard intervention for ATAAD. However, management of ATAAD in the setting of malperfusion syndrome, particularly mesenteric malperfusion, remains unsettled. Some have proposed a staged management approach that initially prioritizes reperfusion of critically malperfused end organs in prioritized via endovascular techniques, such as stenting or fenestration, followed by delayed open repair.^3^^,^^4^

We present the case of a 62-year-old man with ATAAD and concomitant mesenteric malperfusion syndrome who underwent stenting of the descending aorta first, then required an ascending aortic stent as a temporizing measure to allow for resolution of malperfusion and bridge to open repair. Written informed consent for publication of this case and any accompanying images or data was obtained from the patient; institutional review board approval was not required.

## Case Report

A 62-year-old man with an extensive medical history presented to an outside hospital with abdominal pain, nausea, hematemesis, and hematochezia for 1 day. An initial noncontrast computed tomography of the abdomen was unremarkable. Further workup revealed severe gastritis, esophagitis, and multiple gastric and duodenal ulcers on esophagogastroduodenoscopy (EGD). A computed tomography with contrast image showed a Stanford TAAD extending from the aortic root to the common iliac arteries, with evidence of visceral malperfusion and near-complete collapse of the true lumen from which all major abdominal vessels originated (Video 1). From initial presentation at the outside hospital to diagnosis of ATAAD was 5 days. The patient was then transferred to our center for care on the same day.

On arrival, the patient endorsed abdominal discomfort with tenderness to palpation and guarding on exam. He had evidence of acute liver and kidney injury. Due to severe ongoing mesenteric malperfusion syndrome, he was deemed too high risk for immediate open repair. Upon presentation, his laboratory values showed the following: lactate of 5.6 mmol/L, aspartate aminotransferase of 1340 units/L, alanine aminotransferase of 3021 U/L, international normalized ratio of 1.8, and serum creatinine of 1.53 mg/dL. Given the patient's recent EGD at the outside hospital, we did not believe that a laparotomy was necessary. The primary goal on arrival of the patient was to resolve the malperfusion. The decision was made to proceed with a staged intervention, prioritizing resolution of visceral malperfusion via an endovascular approach. The time between the initial EGD at the outside hospital to first operative intervention was 2 days.

**Figure 1:**
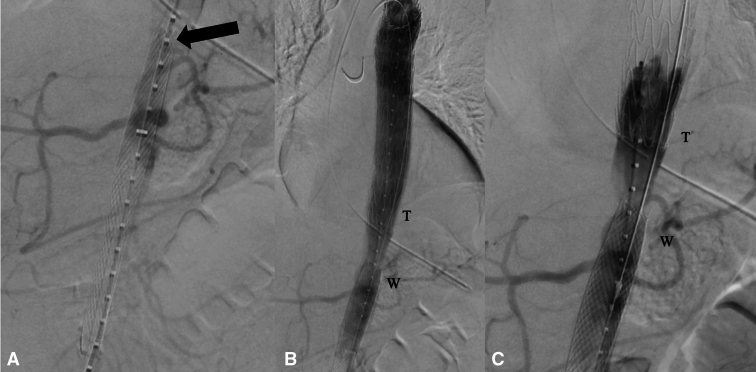
Aortogram of the descending thoracic aorta. A, The arrow indicates proximal collapse of the Wallstent (Boston Scientific). B, Thoracic endovascular aortic repair + Wallstent with true lumen compression in the intervening segment. C, Magnified image of true lumen compression.

Intravascular ultrasound (IVUS), via right common femoral access, was used to ensure true lumen access. The malperfusion was dynamic. Angiography was utilized to assess patency of the critical blood vessels. It demonstrated diminished flow with no thrombosis through the superior mesenteric artery and celiac trunk. The sizes of the aorta at the sinotubular junction (STJ), zone 1, zone 3, and visceral segments were 38, 36, 35, and 22 mm, respectively. We oversized our endografts between 0 and 10%. A 24 × 70 mm Wallstent (Boston Scientific) was deployed from zone 5 to zone 9 of the aorta. The Wallstent graft was chosen for placement in zones 5 through 9 because of its radial strength, in the hopes that we get expansion of the true lumen; however, because of the high flow of the false lumen, there was collapse of the proximal Wallstent segment (Figure 1, *A*). We did not consider using other open cell stent grafts because they do not provide adequate radial force to overcome the false lumen pressure. The disadvantage of the Wallstent is that it does not allow for future endovascular procedures, but no static malperfusion was encountered in the visceral arteries. Balloon dilation was attempted several times; however, the proximal Wallstent segments remained collapsed. A 37 × 20 mm cTAG (W.L. Gore & Associates) was then deployed in zone 3. The endograft expanded well; however, repeat aortogram and IVUS demonstrated true lumen collapse in the segment between the distal end of the endograft and proximal Wallstent (Figure 1, *B* and *C*). A 37 × 10 mm cTAG was then deployed from the distal portion of zone 3 to zone 5. Repeat IVUS demonstrated good expansion of all 3 devices. We do not have the IVUS recording of the visceral segment.

**Figure 2:**
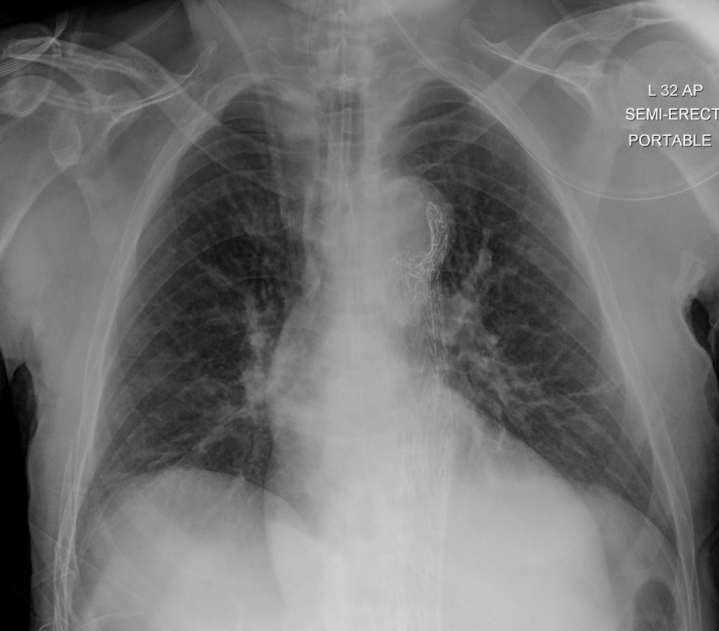
Postoperative day 1 chest radiograph demonstrating collapse of proximal segment of thoracic endovascular aortic repair stent.

Postoperatively, visceral organ perfusion initially improved with decreasing transaminases, creatinine, and lactate levels. However, the initial chest radiograph 2 hours postoperatively showed collapse of a proximal thoracic endovascular aortic repair segment (Figure 2). The decision was made to return to the operating room and stent the ascending aorta to depressurize the false lumen. With imaging showing the tear near the STJ, it was understood that there would likely be a Type 1A endoleak. With rapid ventricular pacing and dropping the mean arterial pressure to 40 mm Hg, a 40 × 10 mm cTAG graft was positioned into the ascending aorta from the STJ to the innominate artery (Video 2). During the operation, we were prepared for sternotomy and open repair during zone 0 deployment in the event there was aortic rupture.

**Figure 3:**
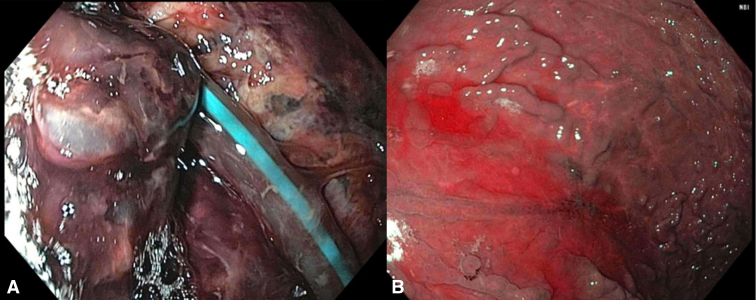
Endoscopic images of gastric mucosa. A, Initial hemorrhagic gastric mucosa. B, Healed gastric mucosa after intervention.

Subsequently, the patient's end-organ function continued to improve, and a repeat endoscopy revealed healed gastric mucosa (Figure 3). The patient was discharged to rehabilitation on postoperative day 28 to allow for recovery of his physical deconditioning and nutritional status. After discharge and before elective open repair, the blood pressure goal was between 90 and 130 mm Hg systolic and 60 and 80 mm Hg diastolic.

Three months later, the patient returned as an outpatient for elective ascending stent graft explant and to reconstruct the aortic root with total arch replacement. The timing of open repair was 3 months after discharge because the patient was very deconditioned during his initial presentation and the goal was to ensure that the patient would tolerate this surgery by rehabilitating adequately before surgery.

During the operation, the patient was cannulated centrally using epiaortic ultrasound with placement of the arterial cannula in the true lumen of the aortic arch distal to the stent graft. The innominate artery and left carotid artery were debranched using a trifurcated graft to allow for bilateral antegrade cerebral perfusion. Systemic circulatory arrest was then done at 22 °C, and the previous graft was explanted with a longitudinal ascending aortotomy (Video 3). A Thoraflex anteflow (Terumo Aortic) device was deployed. The distal anastomosis was sewn to the cuff followed by the proximal anastomosis to the trifurcate. We then connected the third limb of the trifurcated graft to the left subclavian artery. The patient's postoperative course was unremarkable and he was discharged home on postoperative day 14.

## Comments

An aortic dissection involves separation of the intimal and medial layers of the aortic wall, creating a true and false lumen. In this case, dynamic malperfusion occurred due to collapse of the true lumen from high-pressure flow in the false lumen, ultimately leading to compromised visceral perfusion. With dynamic malperfusion, the dissection flap acts as a 1-way valve, intermittently occluding blood flow. This pathophysiology directly informed our approach. The initial descending aortic stenting aimed to restore flow to visceral organs by expanding the true lumen, but persistent pressurization of the false lumen required urgent ascending stent grafting to seal the primary tear.

This case demonstrates the feasibility of an ascending stent graft to manage mesenteric malperfusion syndrome while delaying open aortic repair. Stenting the ascending aorta during the initial operation could have treated the malperfusion; however, significant concern remained that we would not achieve a seal given location of the primary tear in the ascending aorta. This was our rationale on why we stented the descending aorta first. Initially stenting the ascending aorta alone may have been sufficient, but with a Type 1A endoleak, the risk of collapse of the false lumen distally requiring additional stenting remained.

There is an increasing body of evidence suggesting that an endovascular-first approach to acute dissection with mesenteric malperfusion has improved short- and long-term outcomes.^5^ A Michigan group pioneered the reperfusion first concept in 1996 and recently reported 20-year long-term outcomes showing that patients who had delayed central repair after resolution of mesenteric malperfusion syndrome had short- and long-term survival similar to patients without malperfusion syndrome (2.1% vs 7.5%, respectively; *P* = .5).^5^

Endovascular stent graft therapy for ascending aorta and aortic root pathology is challenging due to several anatomic and hemodynamic factors. The ascending aorta is a curved with an outer curvature much longer than the inner curvature. This makes stent sizing, deployment, and fixation difficult.^E1^ Additionally, the ascending aorta is short (∼10 mm), exposed to high velocity blood flow, and has varying mobility and compliance across the cardiac cycle.^E2^ This environment places high displacement forces on an ascending aortic stent graft.

It is important to acknowledge that ascending aorta stent grafts may not be feasible in several important clinical scenarios. Patients with significantly dilated aortic roots present a major challenge because current stent graft technology is not designed to accommodate the curvature, expansile nature, and short segment of the aortic root. In such cases, achieving an adequate seal is difficult, increasing the risk of stent migration and Type 1A endoleak, and the proximal tear often cannot be excluded. Additionally, patients with acute aortic valve insufficiency or coronary malperfusion are not candidates for this approach. Dissection involving the aortic root can lead to severe regurgitation, and involvement of the coronary ostia can result in myocardial ischemia, both of which require direct surgical correction. An endovascular stent would not resolve these life-threatening issues and could delay appropriate intervention. Similarly, patients presenting with cardiac tamponade due to pericardial rupture are not suitable for endovascular management. These patients need emergency surgical decompression and repair because endovascular stenting cannot adequately seal the root or control hemorrhage in this context.

Another important discussion point of this case is ballooning the aorta with an uncovered (bare metal) stent in the setting of AAD. This innately carries both significant benefits and serious risks, particularly when used to manage malperfusion syndromes such as mesenteric ischemia, as shown in those utilizing the stent-assisted balloon-induced intimal disruption and relamination (STABILISE) technique. Among the primary benefits is the ability to rapidly re-expand the true lumen. In this case, ballooning and stenting allowed for improved perfusion and resolution of end-organ dysfunction and served as a bridge to delayed definitive open repair once the patient was more stable.

The acutely dissected aortic wall is fragile and balloon expansion carries the risk of aortic rupture, which is often fatal. There is also a risk of propagating the dissection flap, potentially worsening the patient's condition or compromising additional branch vessels. Furthermore, uncovered stents do not address the proximal entry tear, which can result in Type 1 endoleaks and continued pressurization of the false lumen, complicating subsequent repairs.

Endovascular stent graft interventions for ascending aorta and aortic root pathologies are the last frontier of the endovascular revolution. Ascending aortic stent grafts are poised to add to the armamentarium of cardiac surgeons managing ATAAD in their patients. This case report describes the novel use of an ascending aortic stent graft to seal and temporize a proximal entry tear in a patient presenting with mesenteric malperfusion syndrome. The patient presented here was safely bridged to open aortic repair after several months, with safe explanation of the ascending aortic stent graft.

## Conflict of Interest Statement

The authors reported no conflicts of interest.

The *Journal* policy requires editors and reviewers to disclose conflicts of interest and to decline handling or reviewing manuscripts for which they may have a conflict of interest. The editors and reviewers of this article have no conflicts of interest.

## Supplementary Data

Video 1 Computed tomography angiography showing Stanford type A dissection with true lumen collapse and visceral malperfusion. Video available at: https://www.jtcvs.org/article/S2950-6050(25)00030-0/fulltext.

Video 2 Thoracic endovascular aortic repair extension from sinotubular junction to innominate artery to decompress the false lumen and restore true lumen flow. Video available at: https://www.jtcvs.org/article/S2950-6050(25)00030-0/fulltext.

Video 3 Open graft explant surgical video. Video available at: https://www.jtcvs.org/article/S2950-6050(25)00030-0/fulltext.
