# Supplementary figures and images for: Ascending stent in acute type A as a bridge to open repair
Source: JTCVS Struct Endovasc. 2025 Sep 10;8:100071. doi: 10.1016/j.xjse.2025.100071 (PMC13244772; doi:10.1016/j.xjse.2025.100071)

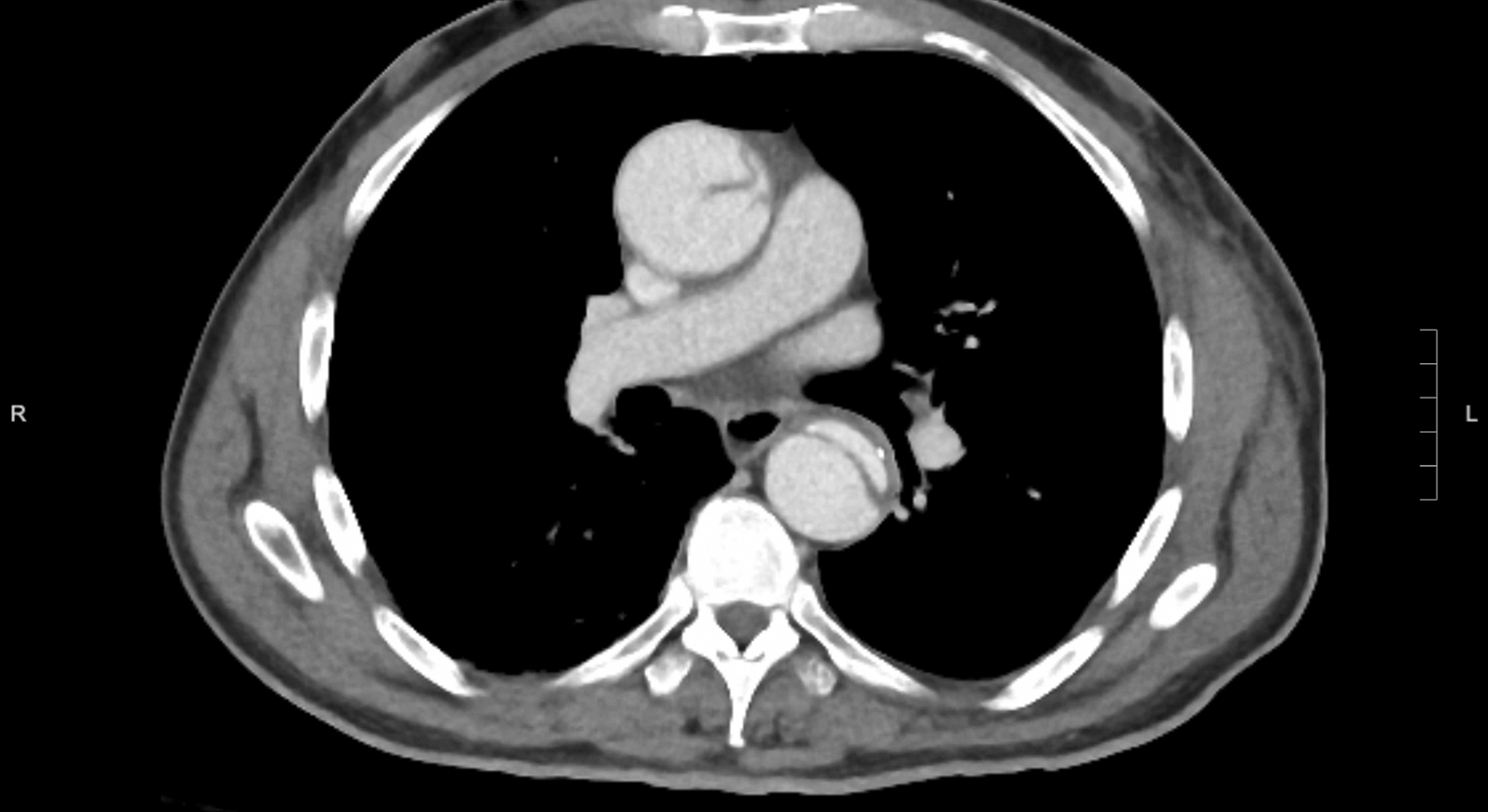

Supplement: Video 1 — Computed tomography angiography showing Stanford type A dissection with true lumen collapse and visceral malperfusion. Video available at: https://www.jtcvs.org/article/S2950-6050(25)00030-0/fulltext. [file fx2.jpg]

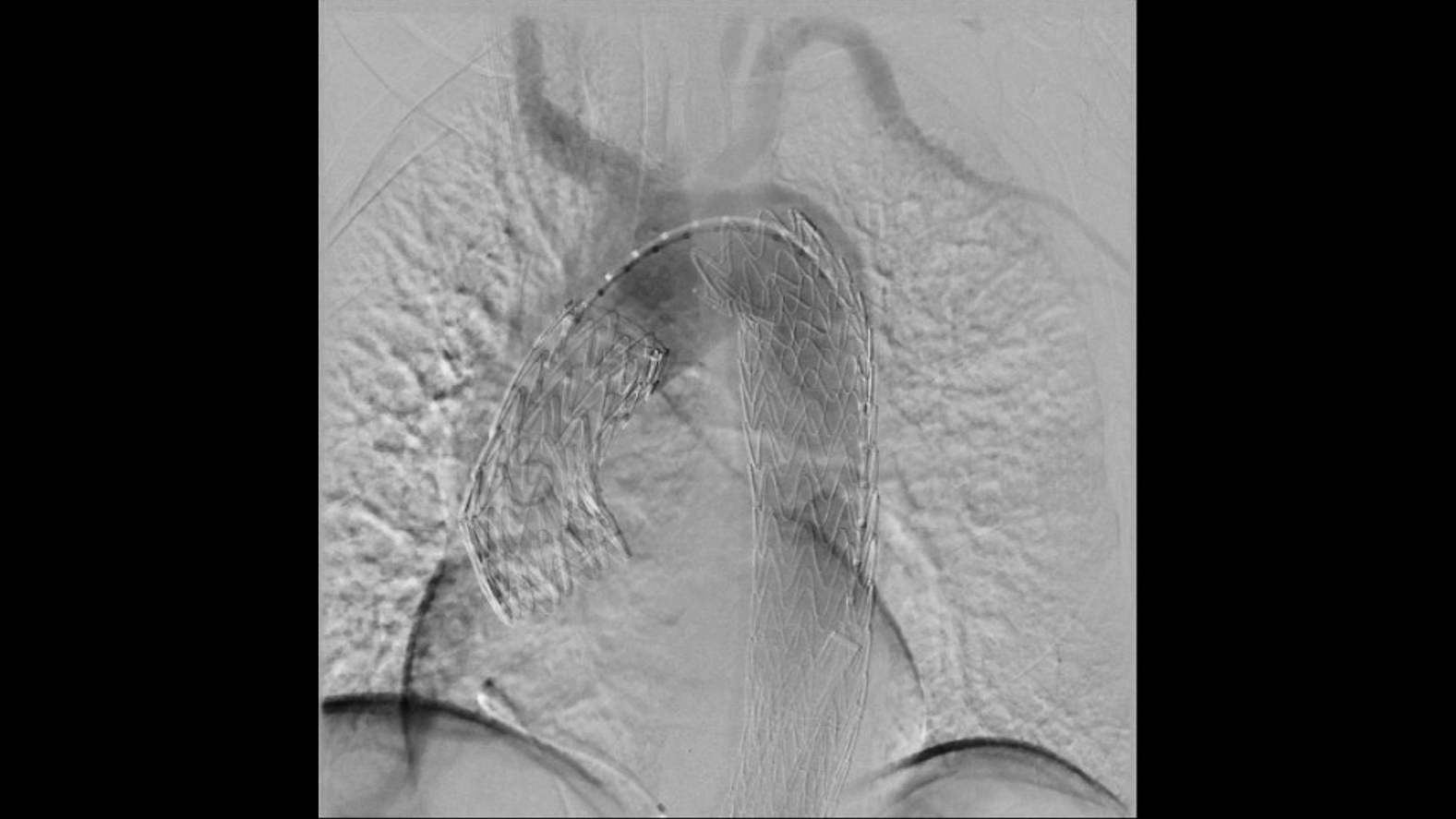

Supplement: Video 2 — Thoracic endovascular aortic repair extension from sinotubular junction to innominate artery to decompress the false lumen and restore true lumen flow. Video available at: https://www.jtcvs.org/article/S2950-6050(25)00030-0/fulltext. [file fx3.jpg]

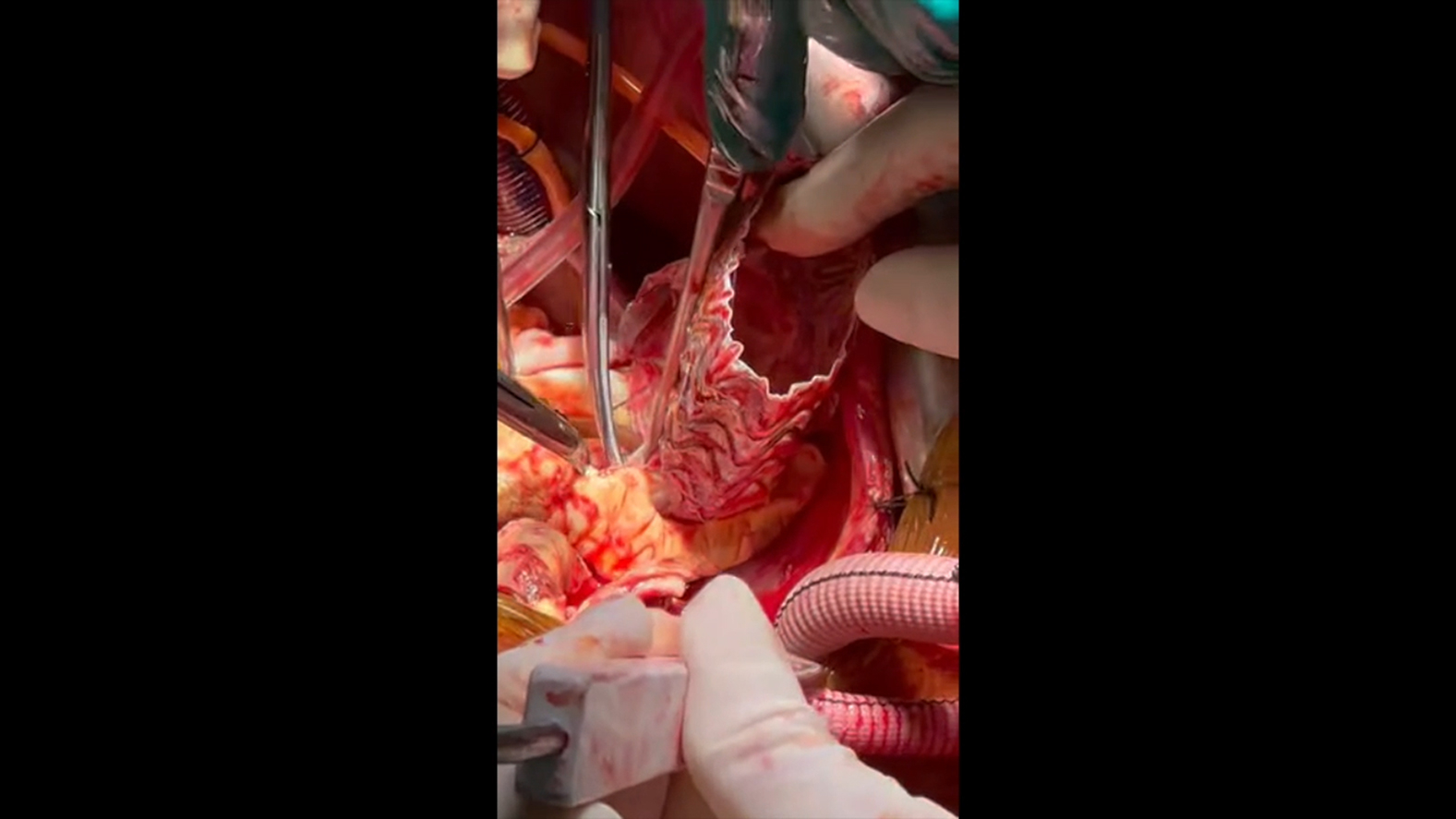

Supplement: Video 3 — Open graft explant surgical video. Video available at: https://www.jtcvs.org/article/S2950-6050(25)00030-0/fulltext. [file fx4.jpg]
